# Sexual Functioning and Impact on Quality of Life in Patients with Early-Onset Colorectal Cancer: A Systematic Review

**DOI:** 10.3390/diseases12040066

**Published:** 2024-03-26

**Authors:** Catalin Vladut Ionut Feier, Ionut Andrei Paunescu, Alaviana Monique Faur, Gabriel Veniamin Cozma, Andiana Roxana Blidari, Calin Muntean

**Affiliations:** 1 First Discipline of Surgery, Department X-Surgery, “Victor Babes” University of Medicine and Pharmacy, 300041 Timisoara, Romania; catalin.feier@umft.ro; 2 First Surgery Clinic, “Pius Brinzeu” Clinical Emergency Hospital, 300723 Timisoara, Romania; 3 Department of Urology, “Pius Brinzeu” Clinical Emergency County Hospital, 300736 Timisoara, Romania; 4 Faculty of Medicine, “Victor Babes” University of Medicine and Pharmacy, 300041 Timisoara, Romania; alaviana.faur@student.umft.ro; 5 Department of Surgical Semiology, Faculty of Medicine, “Victor Babeş” University of Medicine and Pharmacy Timisoara, 300041 Timişoara, Romania; gabriel.cozma@umft.ro; 6 Research Center of Thoracic Surgery, “Victor Babeş” University of Medicine and Pharmacy Timisoara, 300041 Timisoara, Romania; 7 Oncology, Department IX-Surgery, “Victor Babes” University of Medicine and Pharmacy, 300041 Timisoara, Romania; andiana.blidari@umft.ro; 8 Medical Informatics and Biostatistics, Department III-Functional Sciences, “Victor Babes” University of Medicine and Pharmacy, 300041 Timisoara, Romania; cmuntean@umft.ro

**Keywords:** quality of life, sexual dysfunction, colorectal cancer

## Abstract

This systematic review investigates the intersection of early-onset colorectal cancer (EOCRC), sexual functioning, and associated quality of life (QoL), aiming to understand the comprehensive impact of EOCRC on these critical dimensions. Through an extensive search across PubMed, Scopus, and Embase up until November 2023, this study synthesized evidence from the literature while adhering to PRISMA guidelines. The studies included EOCRC patients under 50 years, which examined sexual functioning and QoL using validated instruments, and were published in English. After a rigorous screening process, five relevant studies were identified from an initial pool of 2184 articles. This review includes data from five studies involving 2031 EOCRC patients. The key findings revealed a high prevalence of sexual dysfunction, with up to 50% of men experiencing impotence and 58% reporting sexual dysfunction, alongside 36% of women in some studies. Pain was described by 12% to 31% of patients. Anxiety and depression were notably prevalent, affecting up to 69% of participants. EOCRC profoundly impacts sexual functioning and QoL, with a significant prevalence of sexual dysfunction and psychological distress among affected individuals. These findings suggest the need for oncological management strategies that include not only medical treatment but also psychological support and sexual health interventions. This systematic review emphasizes the importance of holistic patient care approaches, advocating for further research and clinical attention to address the complex needs of younger EOCRC patients.

## 1. Introduction

The incidence of colorectal cancer (CRC) among younger adults has been on the rise globally, presenting unique challenges in management and survivorship [1,2]. Traditionally considered a disease of older adults, the increasing trend of early-onset colorectal cancer (EOCRC), defined as CRC diagnosed in individuals under the age of 50, has garnered significant attention in the medical community [3,4]. Epidemiological studies have indicated a steady increase in EOCRC prevalence across diverse populations, suggesting the need for focused research on its implications. The American Cancer Society reports a 2% annual increase in the incidence of EOCRC in the United States, suggesting a shift in the age-related risk landscape of this malignancy [5,6]. This demographic shift raises concerns regarding the psychosocial, physical, and sexual well-being of younger patients, who are often in the midst of career building, family life, and social development [7].

Quality of life (QoL) in cancer survivors has emerged as a paramount consideration in oncology care, extending beyond traditional outcomes such as survival rates [8]. In patients with EOCRC, QoL issues are particularly pronounced due to the potential for more aggressive disease, the need for intensive treatment, and the broader impact on an individual’s life trajectory [9]. Studies have shown that colorectal cancer survivors experience significant challenges in physical functioning, emotional well-being, and social integration [10,11]. Moreover, the adverse effects of cancer treatments, such as chemotherapy, radiation, and surgery, can exacerbate these challenges, leading to long-term implications for patients’ quality of life [12,13,14].

Sexual functioning is a critical aspect of quality of life that is often negatively impacted in cancer survivors [15]. The anatomical and physiological changes resulting from surgical and non-surgical treatments can lead to sexual dysfunction, affecting both men and women [16]. For men, issues may include erectile dysfunction and ejaculatory problems, whereas women may experience vaginal dryness, dyspareunia, and infertility [17,18]. These sexual health issues are not only detrimental to personal relationships and psychological well-being but also contribute to the overall burden of cancer survivorship. Despite its significance, sexual functioning remains under-addressed in the clinical management of colorectal cancer patients.

The intersection of EOCRC, quality of life, and sexual functioning presents a complex array of challenges for patients, healthcare providers, and researchers. Understanding the impact of EOCRC on these dimensions is essential for developing comprehensive care strategies that address the holistic needs of younger patients. This systematic review aims to synthesize current evidence on the sexual functioning and associated quality of life in patients with EOCRC, offering insights into the prevalence, contributing factors, and potential interventions to support this unique patient population.

## 2. Materials and Methods

### 2.1. Protocol and Registration

To ensure a thorough and methodical examination of the literature, this study implemented an elaborate search strategy across several key electronic databases, including PubMed, Scopus, and Embase. The search was designed to include literature published up until November 2023, capturing the most recent studies available on the topic. The focus of the search strategy was to collate literature pertaining to the impact of EOCRC on sexual functioning and quality of life.

The search strategy incorporated a broad array of keywords and phrases relevant to the study’s objectives, emphasizing the assessment of sexual functioning and quality of life in patients with EOCRC. Key search terms included: “early-onset colorectal cancer”, “colorectal neoplasms”, “sexual health”, “sexual dysfunction”, “quality of life”, “QoL”, “patient-reported outcomes”, “health-related quality of life”, “HRQoL in colorectal cancer”, “impact of cancer treatment on sexual functioning”, “survivorship and sexual health”, “fertility issues in cancer patients”, “psychological well-being”, “emotional distress”, “body image”, “intimacy and relationships”, “erectile dysfunction”, “vaginal dryness”, “dyspareunia”, “sexual counseling in cancer care”, and “interventions for sexual dysfunction”.

Boolean operators were employed to effectively refine and combine the search terms. The search string was structured as follows: (“early-onset colorectal cancer” OR “young colorectal cancer patients” OR “EOCRC”) AND (“sexual health” OR “sexual functioning” OR “sexual dysfunction” OR “fertility issues”) AND (“quality of life” OR “QoL” OR “patient-reported outcomes” OR “health-related quality of life”) AND (“psychological well-being” OR “emotional distress” OR “body image”) AND (“intimacy and relationships” OR “erectile dysfunction” OR “vaginal dryness” OR “dyspareunia”) AND (“sexual counseling” OR “interventions for sexual dysfunction” OR “supportive care in oncology”).

The Preferred Reporting Items for Systematic Reviews and Meta-Analyses (PRISMA) guidelines were followed [19]. This systematic review protocol ensures that the methodology is structured, transparent, and reproducible. To further enhance the transparency and accessibility of our research process and findings, this review has been registered with the Open Science Framework (OSF), facilitating open access to our methodology and outcomes, with the registration code osf.io/tc4r6. This comprehensive search strategy aimed to capture a wide spectrum of studies, allowing for a nuanced understanding of the impact of EOCRC on sexual functioning and quality of life, thereby informing future research and clinical practice in this area.

### 2.2. Eligibility Criteria and Definitions

The eligibility criteria were designed to identify studies that provide insights into how EOCRC affects sexual functioning and overall quality of life. Therefore, this review considered the following inclusion criteria: (1) Study population: the studies must include patients diagnosed with EOCRC, defined as colorectal cancer diagnosed in individuals under the age of 50. (2) Focus on sexual functioning and quality of life: the research must specifically examine the impact of EOCRC on sexual functioning, as the main study outcome, and include the impact on quality of life. This includes studies assessing sexual health, sexual dysfunction, fertility issues, psychological well-being, body image, intimacy and relationships, and interventions aimed at addressing sexual health concerns. (3) Types of studies: inclusion of a wide range of study designs, including randomized controlled trials (RCTs), observational studies, clinical trials, cohort studies, case–control studies, and cross-sectional studies. The studies should provide clear and detailed methodologies regarding the assessment of sexual functioning and quality of life in EOCRC patients. (4) Outcome measures: studies that utilize validated instruments or clearly defined parameters to assess sexual functioning and quality of life. This can include patient-reported outcome measures (PROMs), quality of life assessments, and specific tools designed to evaluate sexual health and dysfunction. (5) Language: only peer-reviewed articles published in English were included to ensure the feasibility of thorough review and analysis.

The exclusion criteria comprised the following: (1) Non-human studies: research that did not involve human participants, such as in vitro or animal model studies, was excluded to focus solely on human patient experiences and outcomes. (2) Broad cancer focus: studies that did not specifically examine patients with EOCRC, or those that did not differentiate the impact of cancer on sexual functioning and quality of life from other cancer types, were excluded. (3) Lack of specific outcomes: studies that did not provide clear, quantifiable outcomes related to sexual functioning and quality of life, or lack sufficient detail for a comprehensive analysis, were excluded. (4) Grey literature: to maintain the credibility and reliability of the data included in this review, grey literature, including non-peer-reviewed articles, preprints, conference proceedings, general reviews, commentaries, and editorials, were excluded.

### 2.3. Definitions

In this systematic review, EOCRC was defined as colorectal cancer diagnosed in individuals under the age of 50. This definition aligns with emerging research trends and clinical observations indicating a distinct epidemiological pattern and clinical presentation in younger patients compared to their older counterparts [4]. The choice of this age cutoff was based on the intention to explore and identify the specific challenges and outcomes faced by this particular demographic group.

Quality of life assessment was considered through the use of standardized surveys and patient self-reported feedback in order to capture a broad spectrum of patient experiences and outcomes related to physical, emotional, social, and sexual well-being. Standardized surveys were identified as key instruments due to their validated nature and widespread use in oncology research. These tools are designed to quantify various dimensions of QoL in a reliable and sensitive manner. Patient self-reported feedback was considered as complementary or independent to these standardized measures, offering deeper insights into the personal and subjective aspects of living with EOCRC.

### 2.4. Data Collection Process

**Figure 1:**
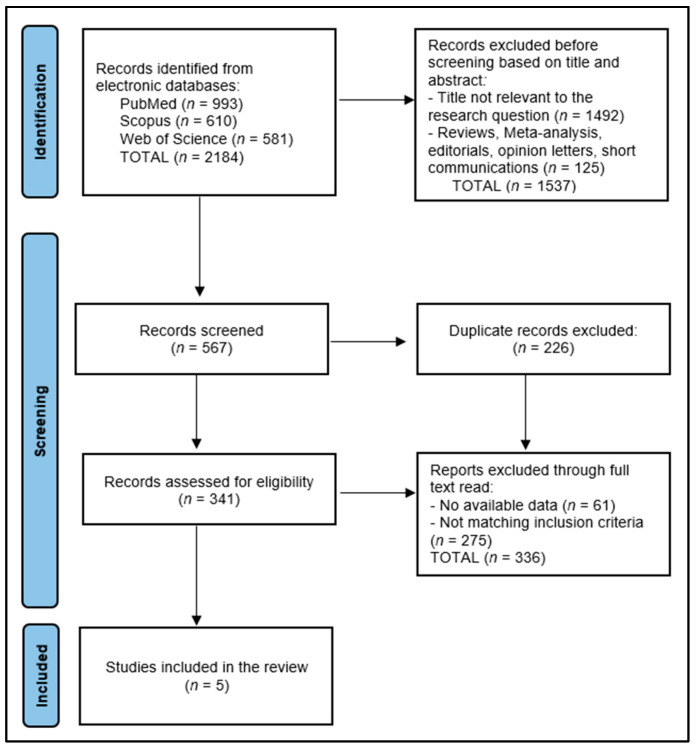
PRISMA Flow Diagram.

The data collection process for this systematic review commenced with the removal of 226 duplicate entries, followed by a rigorous screening of 567 abstracts by two independent reviewers to assess each study’s relevance based on predefined inclusion and exclusion criteria. This step ensured that only studies specifically focusing on sexual functioning and quality of life in EOCRC patients were considered. Discrepancies between reviewers were resolved through discussion or, if necessary, consultation with a third reviewer to achieve consensus. The initial database search yielded 2184 articles, from which five relevant studies were identified for inclusion in the final study. This careful selection process aimed to ensure that the final pool of studies included in this review was of high relevance and quality, thereby providing a comprehensive overview of the impact of EOCRC on patients’ sexual health and overall quality of life, as presented in Figure 1.

### 2.5. Risk of Bias and Quality Assessment

For the assessment of study quality, our review was evaluated using the Newcastle–Ottawa Scale [20], a widely recognized tool that assesses three critical dimensions: the selection of study groups, the comparability of these groups, and the ascertainment of either the exposure or outcome of interest for case–control or cohort studies, respectively. Each study is awarded stars in these categories, cumulating in a score that classifies the study quality as either low, medium, or high in order to facilitate a nuanced evaluation of study quality, enabling the systematic identification of research that meets high methodological standards. To ensure the objectivity and reproducibility of our quality assessment process, each study was independently evaluated by two researchers. Discrepancies in quality assessment scores were resolved through discussion, or if necessary, consultation with a third researcher.

## 3. Results

### 3.1. Study Characteristics

The systematic review analyzed a total of five studies [21,22,23,24,25], as delineated in Table 1. These studies originated from diverse geographic locations, including the United States [21,22,25], Israel [23], and Ireland [24], and were conducted between 2014 and 2023. The research methodologies employed across these studies varied, encompassing both cross-sectional and prospective cohort designs. Specifically, Bailey et al. [21] and Perl et al. [23], as well as Stal et al. [25], utilized a cross-sectional approach to capture a snapshot of the impact of colorectal cancer on sexual health and quality of life at a single point in time. Conversely, Sanford et al. [22] and REACCT [24] conducted prospective cohort studies, providing insights into the evolution of these impacts over time. These study designs reflect a balanced mix of methodologies, offering both immediate and longitudinal perspectives on the subject matter. The quality of the studies, as evaluated in the review, varied, with Bailey et al. [21] receiving a low-quality rating, whereas the rest were considered to be of medium quality.

### 3.2. Patients’ Characteristics

The findings from Table 2 offer a detailed examination of patient characteristics in the five studies involving EOCRC, encompassing a total of 2031 patients. The average age of participants across the studies varied slightly but was below 50 years, as per the definition of EOCRC. Specifically, ages ranged from 34.3 years in the study conducted by Sanford et al. [22] to 43.4 years in the study conducted by Bailey et al. [21], indicating the early-onset nature of CRC among the studied populations. The gender distribution was relatively balanced in all the studies, with a slight male predominance in three studies (Bailey et al. with 50.7% men, the REACCT collaboration with 57% men, and Stal et al. with 61.9% men) and a female predominance in one study (Perl et al. [23] with 55% women), reflecting the varied impact of EOCRC across genders.

Ethnicity and race data, provided in three of the studies, revealed a majority of white participants, with Bailey et al. [21] reporting 80.1%, Sanford et al. [22] reporting 83.7%, and Stal et al. [25] reporting 77.9% white participants. This demographic information suggests a potential focus on predominantly white populations in EOCRC research, with limited representation from other racial and ethnic groups. The comparison groups varied across the studies, including comparisons based on age (late-onset CRC vs. EOCRC in the study conducted by Bailey et al. [21]), gender (male vs. female in the studies conducted by Perl et al. [23] and Stal et al. [25]), and no specific comparison was reported in the REACCT study [24].

### 3.3. Disease Characteristics

Table 3 presents a detailed comparison of disease characteristics across the five studies, showcasing the diversity in disease duration, severity, surgical history, treatments undertaken, and complications among patients with EOCRC. The disease duration reported varied significantly, with Bailey et al. [21] not specifying a duration, whereas the REACCT collaboration [24] and Stal et al. [25] reported disease durations of 42 months and 32.7 months, respectively. In the study conducted by Bailey et al. [21], the disease duration was 10.6 years. The variability in disease duration before study enrollment may impact treatment outcomes and complicates the comparison of results across studies.

Disease severity, indicated by the proportion of patients with metastatic disease or classified as Stage IV, varied widely across the studies. For instance, Bailey et al. [21] reported metastasis in 14.5% of patients, whereas the REACCT study [24] and Stal et al. [25] reported Stage IV disease in 12.8% and 2.6% of patients, respectively. Surgical history was a common thread among the studies. A high percentage of patients underwent surgery, which was accompanied by ostomy in 15.6% of patients from Bailey et al.’s study [21], 43% of patients in the REACCT collaboration [24], and 35.7% of patients in the study conducted by Stal et al. [25]. The prevalence of surgery and ostomy reflects the aggressive nature of treatment strategies for EOCRC, which aim to remove the tumor and prevent the spread of disease. However, the significant physical and psychological impacts of these interventions, particularly ostomy, on patients’ quality of life cannot be overstated, emphasizing the need for comprehensive post-operative care and support.

Patients underwent chemotherapy in 86.2% of cases in Bailey’s study [21], and as low as 6% of cases in the study conducted by Sanford et al. [22]. In the REACCT study, the proportion was similar to that of the study conducted by Stal et el. [25], where 34.1% and 35.0% of patients underwent chemotherapy, respectively. Immunotherapy was reported in only the study conducted by Stal et al. [25], where 27.4% of all patients underwent immunotherapy. However, this study was the most recent (conducted in 2023), so the advancement of medical science must be considered.

Complications, such as active cancer, disease progression, major post-operative complications, and relapse, were reported in the study conducted by Stal et al. [25]. A relapse rate of 61.4% and a disease progression rate of 70.2% were reported in the study conducted by Sanford et al. [22]. These complications show the aggressive nature of EOCRC and the challenges in achieving long-term remission. The high rate of disease progression and relapse is likely associated with familial predisposition among EOCRC patients.

### 3.4. Sexual Function and Quality of Life

Table 4 sheds light on the intricate challenges faced by patients with EOCRC, focusing on sexual function, quality of life, psychometrics, and functional outcomes, as detailed in the studies conducted by Bailey et al. [21], Sanford et al. [22], Perl et al. [23], REACCT [24], and Stal et al. [25]. This analysis reveals the profound impact of EOCRC on both physical and psychological domains, emphasizing the need for a comprehensive care approach.

The studies collectively described significant sexual dysfunction among EOCRC patients, with Bailey et al. [21] reporting a high prevalence of impotence (50%) and sexual dysfunction in both men (58%) and women (36%), alongside anxiety (69.6%) and low body image (81.8%). These findings indicate a considerable burden on sexual health and psychological well-being, necessitating targeted interventions to address these issues. Similarly, Sanford et al. [22] observed a severe impact on intimate life (24%) and emotional distress, including sadness and low mood, further underscoring the emotional toll of EOCRC. Perl et al. [23] noted worsened sexual dysfunction in women, suggesting a gender-specific impact that requires tailored support strategies.

REACCT [24] reported notably lower rates of sexual dysfunction and infertility (4.5% and 1%, respectively), which may reflect methodological differences or the effectiveness of specific treatments, as well as a shorter duration of time since disease onset. However, the broader trend across these studies points to the significant impact of EOCRC on sexual and reproductive health. Stal et al. [25] provided detailed insights into the extent of sexual dysfunction, with low scores on the Female Sexual Function Index and the International Index of Erectile Function.

Beyond sexual health, the studies revealed widespread psychometrics and functional issues, such as anxiety, distress (37.8% in the study conducted by Sanford et al. [22]), micturition problems, and bowel dysfunction (34% of patients in the study conducted by Bailey et al. [21] and 16% in the REACCT collaboration study [24]). The prevalence of pain varied between the studies, with 12% of patients in the study conducted by Bailey et al. [21], 27% of patients in the study conducted by Sanford et al. [22], and 31% of patients in the study conducted by Perl et al. [23] experiencing pain. These outcomes not only affect quality of life but also emphasize the multifaceted nature of the challenges faced by EOCRC patients. The high rates of anxiety (69.6%) and low body image (81.8%) reported by Bailey et al. [21], for instance, suggest the need for comprehensive support services that address mental health and body image concerns.

## 4. Discussion

### 4.1. Summary of Evidence

This systematic review provides a nuanced understanding of EOCRC and its impacts on patients’ sexual health, quality of life, and overall well-being, underlying the unique challenges faced by younger patients regarding their sexual function and QoL. However, the predominance of white participants in the studies included points to a significant gap in the research; the experiences of racial and ethnic minorities with EOCRC are underrepresented, suggesting the need for broadened research studies, as racial and ethnic discrepancies can be considered confounding factors for disease evolution and QoL.

The examination of disease characteristics, such as duration, severity, and treatment outcomes, revealed the aggressive nature of EOCRC and its profound impact on patients. The variability in disease duration and severity across the studies complicates the comparison of outcomes, indicating the need for standardized metrics in EOCRC research. The high prevalence of surgery and ostomy, alongside the utilization of chemotherapy and, in some cases, immunotherapy, demonstrates the intensive nature of EOCRC treatment regimens. These treatments, although necessary, come with significant physical and psychological burdens for patients, as evidenced by the reported complications and high rates of disease progression and relapse. This aspect of the findings points to the critical need for comprehensive post-operative care and ongoing support to manage the side effects and emotional distress associated with EOCRC treatment.

The analyzed studies consistently reported significant sexual dysfunction, psychological distress, and diminished QoL among EOCRC patients, with notable differences in the experiences of men and women. The significant emotional toll and the challenges in maintaining intimacy and positive body image call for a holistic care model that includes sexual health and psychological support as integral components, similar to other common cancer types [26,27,28,29]. The disparities in the findings between the studies, particularly regarding rates of sexual dysfunction and infertility, suggest the need for further research to understand these outcomes better and to develop targeted interventions.

The existing literature also reveals the importance of addressing comprehensive care needs among young cancer patients, focusing on sexual health and fertility as critical aspects that are significantly impacted by cancer diagnosis and treatment. Despite the acknowledged need, less than two-thirds of adolescent and young adult patients are informed about the potential infertility risks associated with cancer treatments [30]. McKay et al. highlighted a substantial gap in reproductive health care, revealing that a mere 29% and 40% of patients had documented discussions about sexual health and fertility, respectively, with their healthcare providers [31]. The necessity for attentive reproductive health care is emphasized from diagnosis through to long-term survivorship, extending the consideration of young adult cancer age boundaries to accommodate shifts towards later parenthood [32]. Furthermore, the text brings to light that patients with EOCRC experience higher rates of sexual dysfunction due to CRC-specific treatments, such as pelvic radiation and surgeries [33], presenting unique survivorship challenges that detrimentally affect fertility and sexual health. A significant portion of EOCRC patients report sexual dysfunction, which not only strains relationships but also impacts their sense of self and quality of life [34].

A similar systematic review was published in 2012 by Traa et al. [35], aiming to evaluate the prevalence of sexual dysfunction among CRC patients and to identify treatment-related and sociodemographic factors affecting sexual dysfunction and the quality of sexual life. However, the literature search was conducted between 1990 and 2010, and analyzed data from 82 studies, without making any distinction between EOCRC and older patients above 50 years of age. The findings revealed a wide prevalence of postoperative sexual dysfunction in men, ranging from 5% to 88%, with about half of the women experiencing similar issues. Factors such as preoperative radiotherapy, having a stoma, surgical complications, and higher age were strongly linked to increased sexual dysfunction. These findings demonstrate that no significant changes have been made over the past decade to address this important matter of sexual life in association with QoL.

In the study conducted by Liot et al. [36], involving 72 patients with a mean age of 58 years who underwent colorectal surgery, the findings revealed a notable gender-based difference in postoperative outcomes. Men showed no significant change in sexual function, quality of life, and marital satisfaction after surgery. In contrast, women experienced a decrease in sexual function, as indicated by their Female Sexual Function Index (FSFI) scores, and in relationship satisfaction, based on their Locke–Wallace satisfaction scores, up to 12 months following surgery.

Besides sexual function, QoL in EOCRC patients is also affected by financial constraints. The study conducted by Blum-Barnett et al. [34] focused on financial burden and presented the stark reality that these patients face, not only grappling with the physical aftermath of the disease but also confronting significant socio-economic and emotional hurdles. In this study, employment emerged as a central theme, revealing how career trajectories, lost wages, and the complexities of navigating health insurance profoundly impact survivors’ financial stability and self-identity, as other literature reports [37,38]. Additionally, the emotional and physical side effects of the disease and its treatment require a holistic care approach.

Another important study was designed by Acquati et al. [39] to assess QoL and sexual dysfunction in patients with EOCRC through a controlled survey of at least 60 couples at all CRC stages within the first five years post diagnosis. The trial was designed to use the Dyadic Coping Inventory [40] and the Relationship Concern and Need for Parenthood subscales from the Fertility Problem Inventory [41], whereas emotional functioning and QoL will be assessed using the Patient Health Questionnaire-8 and the Emotions Thermometer [42,43]. Although the results of this trial are not yet available, they will help to uncover the complexities of sexual health in the context of EOCRC.

This systematic review also identified evolving treatment strategies for EOCRC over the past decade, reflecting how these advancements could influence patient outcomes, especially in terms of sexual function. Treatments have become more aggressive and diverse, including chemotherapy, surgery, and the introduction of immunotherapy, which have had a clear impact on patients’ quality of life and complications, such as sexual dysfunction [36]. These treatment modalities, while extending life expectancy and potentially enhancing disease control, have brought the importance of addressing quality of life issues, including sexual health and psychological well-being, to the forefront. This evolution in treatment regimens promotes the necessity for comprehensive care strategies that not only focus on prolonging life but also on improving quality of life for EOCRC patients.

Nevertheless, the systematic review revealed a notable gap in the analyzed studies. Although they documented sexual dysfunction issues in patients with EOCRC, they did not explore interventions for treatment or prevention. Among the potential strategies, phosphodiesterase inhibitors, such as sildenafil, have shown promise in men for enhancing erectile function post-treatment, as highlighted in the study conducted by Kim et al. [44]. This study supports the use of a penile rehabilitation protocol, possibly combining phosphodiesterase inhibitors, vacuum erection devices, and intracorporeal injections, to yield significant benefits. Additionally, addressing hypogonadism and recommending semen cryopreservation before treatments that risk damaging ejaculatory nerve fibers are crucial considerations.

### 4.2. Limitations

The examination of EOCRC in this systematic review reveals heterogeneity in the study methodologies, particularly regarding disease characteristics and the assessment of sexual function and QoL. The discrepancies observed across the studies in the patient populations, treatment modalities, and outcome measurements complicates the synthesis of findings and highlights the challenge in comparing the effectiveness of interventions. For instance, differences in how sexual dysfunction and quality of life are measured, ranging from validated instruments such as the FSFI and IIEF to more general health surveys, make it difficult to draw clear conclusions about the prevalence and severity of these issues among EOCRC patients. This heterogeneity not only impacts the review’s ability to provide a cohesive analysis but also points to a broader issue in the field: the need for standardized research methodologies to accurately assess and address the multifaceted impacts of EOCRC on patients’ lives.

A significant limitation encountered in this review was the inability to conduct a quantitative or semi-quantitative assessment of heterogeneity across the five observational studies, since none of the analyzed studies reported effect sizes. Moreover, although a large number of studies resulted from the initial query in PubMed, Scopus, and Embase, only five studies were eligible for inclusion in the final analysis due to the EOCRC focus of this study and the requirement of these studies to evaluate sexual functioning among these patients. Lastly, one significant limitation of the analyzed studies is the lack of detailed descriptions of the CRC treatment regimens administered, which could substantially influence patient outcomes related to quality of life and sexual function.

## 5. Conclusions

The current findings indicate that EOCRC profoundly impacts patients’ QoL and sexual functioning. It is imperative to develop and implement comprehensive care strategies that not only address the physical aspects of EOCRC but also prioritize psychological support, sexual health, and overall well-being. While highlighting the necessity for integrated patient care that encompasses both oncological treatment and holistic support, our conclusions also underscore the urgent need for research inclusivity and methodological standardization. Addressing the gap in the representation of racial and ethnic minorities, those without insurance, and individuals from geographically or socioculturally isolated areas, alongside the implementation of standardized metrics for evaluating interventions and outcomes, has emerged as a critical avenue for future studies.

## Figures and Tables

**Table 1:** Study characteristics.

Study and Author	Country	Study Year	Study Design	Study Quality
1 [21] Bailey et al.	United States	2014	Cross-sectional	Low
2 [22] Sanford et al.	United States	2014	Prospective cohort	Medium
3 [23] Perl et al.	Israel	2016	Cross-sectional	Medium
4 [24] REACCT	Ireland	2022	Prospective cohort	Medium
5 [25] Stal et al.	United States	2023	Cross-sectional	Medium

**Table 2:** Patient characteristics.

Study Number	Sample Size	Age (Years)	Gender Distribution	Comparison Group	Race/Ethnicity
1 [21] Bailey et al.	282	43.4	143 (50.7%) men 139 (49.3%) women	Late-onset CRC	White: 226 (80.1%) Hispanic: 31 (1.0%) Black: 17 (6.0%)
2 [22] Sanford et al.	37	34.3	19 (51.3%) men 18 (48.6%) women	≥40 years old CRC	White: 31 (83.7%) Black: 6 (16.3%)
3 [23] Perl et al.	50	35.5	24 (45%) men 26 (55%) women	Male vs. female with CRC	Jewish: 22 (64%) Arab: 2 (4%)
4 [24] REACCT	1428	42	816 (57%) men 612 (43%) women	NR	NR
5 [25] Stal et al.	234	34.6	145 (61.9%) men 87 (38.1%) women	Male vs. female with CRC	White: 180 (77.9%) Black: 23 (10.0%) Hispanic: 22 (9.5%)

NR—Not Reported; CRC—Colorectal Cancer.

**Table 3:** Disease characteristics.

Study Number	Disease Duration	Disease Severity	Surgical History	Treatment	Complications
1 [21] Bailey et al.	10.6 years	Metastasis: 41 (14.5%)	Surgery: 276 (97.9%) Ostomy: 44 (15.6%)	Chemotherapy: 243 (86.2%) Radiation: 147 (52.1%)	Active cancer: 24 (8.5%)
2 [22] Sanford et al.	NR	Metastasis: 13 (35.1%)	NR	Chemotherapy: 18 (48%) Radiation: 6 (16.2%)	Disease progression: 26 (70.2%)
3 [23] Perl et al.	NR	Stage IV: 13 (26.0%)	Surgery: 7 (14%)	Chemotherapy: 3 (6%) Chemoradiotherapy: 40 (80%)	NR
4 [24] REACCT	42 months	Stage IV: 184 (12.8%)	Surgery: 1395 (97%) R0 resection: 1212 (84%) Ostomy: 621 (43%)	Chemoradiotherapy: 487 (34.1%) Radiation: 12 (0.8%)	Major post-operative complication (Clavien Dindo 3–4)
5 [25] Stal et al.	32.7 months	Stage IV: 6 (2.6%)	Surgery: 124 (53.0%) Ostomy: 82 (35.7%)	Chemotherapy: 82 (35.0%) Radiation: 133 (56.8%) Immunotherapy: 64 (27.4%)	Relapse: 143 (61.4%)

NR—Not Reported.

**Table 4:** Sexual function and quality of life.

Risk Factors	Sexual Function	Psychometrics	Functional	Survey	Conclusion
1 [21] Bailey et al.	Impotence 50% Dyspareunia 33% Dysfunction men 58% Dysfunction women 36%	Anxiety 69.6% Low body image 81.8%	Micturition problems 29% Bowel dysfunction 34% Pain 12%	EORTC	High prevalence of impotence in EOCRC compared to old patients Worse body image compared to old patients
2 [22] Sanford et al.	Severe impact of intimate life 24%	Distress 37.8% Sadness 24.3% Low mood 40.5%	Pain 27% Diarrhea 16%	SOAPP MDASI	Significantly higher impact on intimate life compared to old patients
3 [23] Perl et al.	Dysfunction men 58% Dysfunction women 61%	Sleeping disorder 32%	Pain 31% Diarrhea 37%	SF-12 CARES	Worse functioning in women compared with men
4 [24] REACCT	Dysfunction 4.5% Infertility 1%	NR	Bowel dysfunction 16% Bladder dysfunction 7%	NR	NR
5 [25] Stal et al.	FSFI mean 14.3 IIEF mean 13.6 Erection hard enough for penetration never/almost never 37.9%	Low/very low confidence 33.1%	NR	FSFI-6 IIEF-5	8 of 10 females reported FSD, almost all males reported ED

NR—Not Reported; EORTC—European Organization for Research and Treatment of Cancer Colorectal Cancer; MDASI—MD Anderson Symptom Inventory; SOAPP—Symptom Outcomes and Practice Patterns Study; SF—Short Form Health Survey; CARES—Cancer Rehabilitation Evaluation System; FSFI—Female Sexual Function Index; IIEF—International Index of Erectile Function; FSD—Female Sexual Dysfunction; ED—Erectile Dysfunction.

## Data Availability

Not applicable.
